# Nuclear Localization of the Transcriptional Regulator MIER1α Requires Interaction with HDAC1/2 in Breast Cancer Cells

**DOI:** 10.1371/journal.pone.0084046

**Published:** 2013-12-20

**Authors:** Shengnan Li, Gary D. Paterno, Laura L. Gillespie

**Affiliations:** Terry Fox Cancer Research Laboratories, Division of BioMedical Sciences, Faculty of Medicine, Memorial University of Newfoundland, St. John's, Newfoundland, Canada; University of Toronto, Canada

## Abstract

MIER1α is a transcriptional regulator that functions in gene repression through its ability to interact with various chromatin modifiers and transcription factors. We have also shown that MIER1α interacts with ERα and inhibits estrogen-stimulated growth. While MIER1α is localized in the nucleus of MCF7 cells, previous studies have shown that it does not contain a nuclear localization signal. In this report, we investigate the mechanism involved in transporting MIER1α into the nucleus. We explored the possibility that MIER1α is transported into the nucleus through a ‘piggyback’ mechanism. One obvious choice is via interaction with ERα, however we demonstrate that nuclear targeting of MIER1α does not require ERα. Knockdown of ERα reduced protein expression to 22% of control, but did not alter the percentage of cells with nuclear MIER1α (98% nuclear with scrambled shRNA vs. 95% with ERα shRNA). Further evidence was obtained using two stable transfectants derived from the ER-negative MDA231 cell line: MC2 (ERα+) and VC5 (ERα-). Confocal analysis showed no difference in MIER1α localization (86% nuclear in MC2 vs. 89% in VC5). These data demonstrate that ERα is not involved in nuclear localization of MIER1α. To identify the critical MIER1α sequence, we performed a deletion analysis and determined that the ELM2 domain was necessary and sufficient for nuclear localization. This domain binds HDAC1 & 2, therefore we investigated their role. Confocal analysis of an MIER1α containing an ELM2 point mutation previously shown to abolish HDAC binding revealed that this mutation results in almost complete loss of nuclear targeting: 10% nuclear vs. 97% with WT-MIER1α. Moreover, double knockdown of HDAC1 and 2 caused a reduction in percent nuclear from 86% to 44%. The results of this study demonstrate that nuclear targeting of MIER1α requires an intact ELM2 domain and is dependent on interaction with HDAC1/2.

## Introduction

MIER1 is a transcriptional regulator identified from a screen for fibroblast growth factor (FGF) early response genes that are activated during mesoderm induction in *Xenopus laevis* embryos [1]. MIER1 has been shown to repress transcription using several distinct mechanisms, including recruitment of HDAC1 [2], inhibition of the histone acetyltransferase activity of CBP [3] and by displacement of Sp1 from its cognate site in the promoter of target genes [4].

The *mier1* gene is highly conserved among species [5], [6] and gives rise to multiple protein isoforms whose structure consists of a common internal region with variable N- and C- termini [5]. The common region contains four acidic stretches, an ELM2 domain and a SANT domain, all of which play a role in transcriptional regulation [1], [2], [4]. Two functional alternate N-termini have been described: one that includes an additional exon (exon 3A) encoding a *bona fide* nuclear export signal (NES; isoform is designated MIER1-3A) [7] and one that does not (designated MIER1). Two distinct C-termini, α and β, have also been characterized. The α sequence contains a classic LXXLL motif for interaction with nuclear receptors and indeed, MIER1α interacts with ERα in breast carcinoma cells [8]. Moreover, regulated overexpression of MIER1α was shown to inhibit estrogen-stimulated growth in these cells [8]. Analysis of MIER1α protein expression in patient biopsies revealed a dramatic shift in subcellular localization, from nuclear to cytoplasmic during progression to invasive breast carcinoma [8]. These data indicate that nuclear MIER1α may play an important role in regulating breast cancer growth and/or progression.

Understanding the mechanism(s) controlling subcellular localization of the α isoform will be important for elucidating its role in breast cancer. We showed previously that inclusion of exon 3A altered the distribution in MCF7 cells, from nucleus to cytoplasm, of the α but not the β isoform [7]. Thus, alternative splicing may be sufficient to shuttle MIER1α out of the nucleus and regulate its corepressor activity. Interestingly, deletion analysis has demonstrated that the β C-terminus contains the only functional nuclear localization signal (NLS) [9], leading to the question of how MIER1α is transported to the nucleus. In this study, we show that nuclear localization of MIER1α in breast carcinoma cells was not through its association with ERα, as one might predict. Instead, it transported to the nucleus through interaction with HDAC1/2.

## Materials and Methods

### Cell lines and culture conditions

The human breast adenocarcinoma cell line, MCF7, was obtained from the American Tissue Culture Collection (ATCC) and cultured in DMEM (GIBCO) containing 10% serum (7.5% calf serum (GIBCO) plus 2.5% fetal bovine serum (GIBCO)) and 1 mM sodium pyruvate (GIBCO). The MC2 and VC5 cell lines were produced by Dr. V.C. Jordan (Georgetown University Medical Center, Washington, DC) and derived by stably transfecting the ER-negative MDA-MB-231 breast carcinoma cell line with wild-type *erα* or empty vector, respectively, as described [10], [11]. MC2 and VC5 cells were maintained in phenol red-free MEM (GIBCO) containing 5% charcoal-dextran treated fetal bovine serum (HyClone), 1% L-glutamine (GIBCO), 6 ng/ml insulin (Invitrogen) and 200µg/ml Geneticin (Invitrogen). All cells were grown a humidified 37°C incubator with 5% CO_2_.

### Plasmids and Constructs

Human *mier1* gene structure, the sequence of its transcripts and the myc-tag vector (pCS3+MT; Dr David Turner, University of Michigan; http://sitemaker.umich.edu/dlturner.vectors/cs2_polylinker_descriptions) containing full-length *mier1α* have been described in [5]. Myc-tagged MIER1α containing a point mutation ^213^W→A in the ELM2 domain (ELM2 mutant) was produced using the QuikChange site-directed mutagenesis kit (Stratagene) and the following primers: 5′-GAT CAG CTC CTG GCG GAC CCT GAG TAC TTA CC-3′ (forward); 5′-GGT AAG TAC TCA GGG TCC GCC AGG AGC TGA TC-3′ (reverse). For the MIER1α deletion constructs, previously described constructs containing amino acids (aa)1–283, aa163–433, aa164–283, aa287–433, aa164–239, aa240–283, aa164–251 or aa164–273 of MIER1α in the Clontech pM vector [2] were digested with EcoRI and the MIER1α insert was ligated into the EcoRI site of a pCS3+MT vector that had been modified to maintain the MIER1 sequence in-frame with the myc-tag. This modified pCS3+MT, renamed pCS4+MT, contains a thymidine (T) inserted upstream of the EcoRI site. All plasmids were prepared using the NucleoBond Endotoxin-free Maxi Plasmid kit (Clontech), according to the manufacturer's instructions. The sequences/mutations were confirmed by automated dideoxynucleotide sequencing of both strands (DNA Sequencing Facility, The Centre for Applied Genomics, The Hospital for Sick Children, Toronto, Canada). Plasmids containing ERα shRNA, HDAC1 shRNA, HDAC2 shRNA or a control scrambled shRNA were purchased from Origene Technologies, Inc.

### Antibodies

The 9E10 anti-myc tag mouse monoclonal antibody was prepared as described in Blackmore *et al.* [3]. The anti-ERα antibody HC-20, anti-HDAC1 antibody H-51 and anti-HDAC2 antibody H-54 were purchased from Santa Cruz Biotechnology Inc. For confocal analysis, Alexa Fluor-488 labeled donkey anti-mouse and Alexa Fluor-647 labeled donkey anti-rabbit were purchased from Jackson ImmunoResearch Laboratories, Inc. HRP-labeled sheep anti-mouse and donkey anti-rabbit antibodies were purchased from GE Healthcare Corp. Anti-β-actin (A5441) was purchased from Sigma-Aldrich Co.

### Transient Transfection

Cells were transfected by electroporation using the Neon® electroporation device (Invitrogen Corp.) and the following settings: 1000 V, 30 ms, 2 pulses for MCF7 or 1400 V, 10 ms, 4 pulses for MC2 and VC5. 3×10^5^ (MCF7) or 2.6×10^5^ (MC2 and VC5) cells were mixed with 0.5 µg myc-tagged plasmid and loaded into a 10 cl tip for electroporation. For the ERα shRNA knockdown experiments, 1.0µg shRNA and 0.5µg myc-tagged plasmid were mixed together with 3×10^5^ MCF7 cells, and then loaded into a 10µl tip for electroporation. After transfection, cells were plated at a density of 4×10^4^/well in Falcon 8-well culture slides (BD BioSciences) for confocal analysis or 3×10^5^/well in 6-well dish for Western blot analysis. For the HDAC1 and 2 double knockdown experiments, 0.8µg of each HDAC shRNA plasmid was used for electroporation; for single knockdowns, the total amount of plasmid transfected was kept constant by adding 0.8µg of scrambled shRNA plasmid. Electroporation and plating was performed as above. Sixteen hours after electroporation, cells were transfected with 0.5µg plasmid encoding myc-tagged MIER1α using Mirus TransIT-LT1 transfection reagent (Medicorp, Inc.) in a 3∶1 ratio of reagent:DNA (v/w), according to the manufacturers' protocol. Transfected cells were cultured for a total of 48 h, then either fixed with 4% paraformaldehyde/PBS for confocal analysis, or solubilized in 400µl of SDS sample buffer (50 mM Tris-Cl pH6.8, 2% SDS, 5% β-mercaptoethanol, 10% glycerol, 0.1% bromophenol blue) for Western analysis.

### Immunofluorescence, Confocal Microscopy and Analysis

Cells were fixed for 10 min with 4% paraformaldehyde and permeabilized with 0.1% Triton X-100/PBS for 5 min. Non-specific sites were blocked with 5% donkey serum in PBS for 1 h before overnight incubation with primary antibodies (1∶200 dilution) at 4°C. Subsequently, the cells were incubated with Alexa Fluor-488 labeled donkey anti-mouse secondary antibody (1∶200) and/or with Alexa Fluor-647 labeled donkey-anti-rabbit (1∶200) for 1 h at RT. Nuclei were counterstained with 2.5µg/ml 4′, 6-diamidino-2-phenylindole (DAPI; Sigma-Aldrich Co.) for 1 h. All slides were mounted in 10% glycerol/PBS. Cells were examined under an Olympus FluoView FV1000 confocal microscope. Fluorescence images were obtained by sequential z-stage scanning in two or three channels (DAPI, Alexa Fluor-488 and/or Alexa Fluor-647); z-stacks were compiled into individual images.

Quantitative analysis of confocal z-stacks was performed using Image J software v1.48 [12], as described in [7]. Briefly, cell outlines were traced and the sum of the pixel values within the outlines for all slices was determined. After subtracting the background, this value was used as the corrected whole cell MIER1 fluorescence. The sum of the pixel values for nuclei was determined in the same way and used as corrected nuclear MIER1 fluorescence. The nuclear value was subtracted from the whole cell value to obtain cytoplasmic MIER1 fluorescence and the corrected fluorescence value in each compartment was plotted as a proportion of the total. 20-30 cells were measured for each sample.

Statistical analysis was performed using a two-sided Fisher's exact test with the Instat v3.0 software program (Graphpad Software, San Diego, CA, USA).

### Co-immunoprecipitation (co-IP) and Western Blot Analysis

Forty-eight hours post-transfection (described above), cells were washed once with 1xPBS and lysed on ice for 30 min in 1xIP buffer (1% Triton X-100, 150 mM NaCl, 10 mM Tris-Cl pH7.4, 10 mM EDTA, 0.02% Sodium Azide, 1 mM PMSF, 1% protease inhibitor cocktail). Cell lysates were passed several times through a 26-gauge needle then centrifuged at 12,000×g for 15 min at 4°C. The supernatants were incubated overnight at 4°C with anti-HDAC1 or anti-HDAC2 antibody pre-bound to Protein A-agarose beads (Pierce Biotechnology). After incubation, the beads were washed six times with ice-cold 1xIP buffer and bound proteins were solubilized in 30µl of 1.5x SDS sample buffer and analyzed by SDS-PAGE-Western.

Western blot analysis was performed as in [13] using 7% SDS-PAGE gels. Transfers were performed using 0.2µm PVDF membranes (Trans-Blot TurboTM Transfer Pack; Bio-Rad) and the Trans-Blot Turbo^™^ system (Bio-Rad) set at 1.3A, 25 V for 7 min. Membranes were stained using a 1∶1000 dilution of primary antibody, 1∶3000 HRP-labeled secondary antibody and Amersham's ECL Plus Western Blotting System (GE Healthcare Corp.). Quantitative analysis of the ERα, HDAC1 and HDAC2 protein bands was performed using Image J software v1.48 [12].

## Results

### Nuclear localization of MIER1α is not dependent on its interaction with ERα

**Figure 1:**
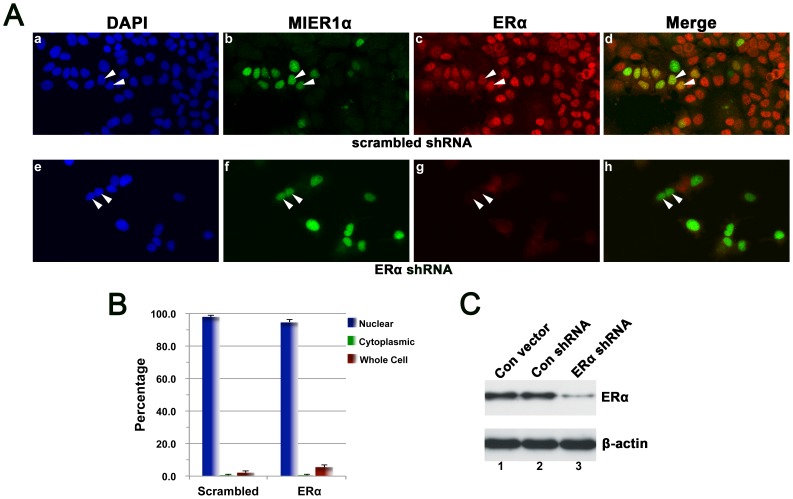
Knockdown of ERα does not affect nuclear localization of MIER1α in MCF7 cells. MCF7 cells were transfected with myc-tagged MIER1α plus either a control, scrambled shRNA or an ERα shRNA and analyzed by confocal microscopy (A, B) or immunoblotting (C). (A) Illustrative examples of cells showing stained nuclei (DAPI; panels a,e), MIER1α localization (9E10 anti-myc tag and an AlexaFluor-488 secondary antibody; panels b, f) and ERα localization (HC-20 antibody and an AlexaFluor-647 secondary antibody; panels c, g). Panels d,h show merged 488 and 647 channels. Arrowheads indicate nuclei. Note that MIER1α is nuclear even in cells that lack detectable ERα (arrowheads in panels f & g). (B) Histogram showing the results of 3 independent experiments; random fields were selected and the staining pattern of each cell within the field was scored visually according to the categories described in the Results. 180-190 cells were scored for each shRNA. Plotted is the percentage of cells in each category ± S.D; there is no significant difference between the percent nuclear for the two samples (p>0.05). (C) Western blot to confirm knockdown of ERα. Extracts from MCF7 cells transfected with myc-tagged MIER1α and either empty vector (lane 1), control scrambled shRNA (lane 2) or ERα shRNA (lane 3). The blot was stained with anti-β-actin (lower panel) to verify equal loading or with anti-ERα (upper panel).

In our original characterization of human MIER1α and MIER1β, we determined that the α isoform localized in the cytoplasm of NIH3T3 cells, while the β isoform was exclusively nuclear [14]. Subsequently, deletion analysis confirmed that MIER1α does not contain a functional NLS [9] and yet it is localized in the nucleus of MCF7 breast carcinoma cells [7]. Given that MIER1α interacts with ERα [8], we investigated whether MIER1α is carried into the nucleus of MCF7 cells by binding to ERα, in a ‘piggyback’ fashion. Cells were transfected with plasmids encoding a myc-tagged MIER1α along with either an ERα shRNA or a scrambled, control shRNA and localization was determined by confocal microscopy. Subcellular localization was scored as: 1) nuclear; if the nucleus was intensely stained, with little or no cytoplasmic staining; 2) cytoplasmic; if staining was primarily in the cytoplasm, with little or no staining in the nucleus; 3) whole cell; if both the nucleus and cytoplasm were stained. The shRNA was effective at knocking down endogenous ERα expression levels, as determined by Western blot and confocal microscopy, while the scrambled shRNA had no effect (Fig. 1A, compare panels c & g; Fig. 1C, compare lanes 2 & 3). ImageJ analysis of the Western blot in Fig. 1C, determined that ERα expression was knocked down to 22% of control. In cells expressing the scrambled shRNA, 98% displayed nuclear MIER1α (Fig. 1A panels a-d, &1B) and this pattern did not change when ERα expression was knocked down. 95% of cells expressing ERα shRNA displayed nuclear MIER1α (Fig. 1A panels e-h, &1B), even cells with no detectable ERα (see arrowheads in Fig. 1A, panels f & g).

**Figure 2:**
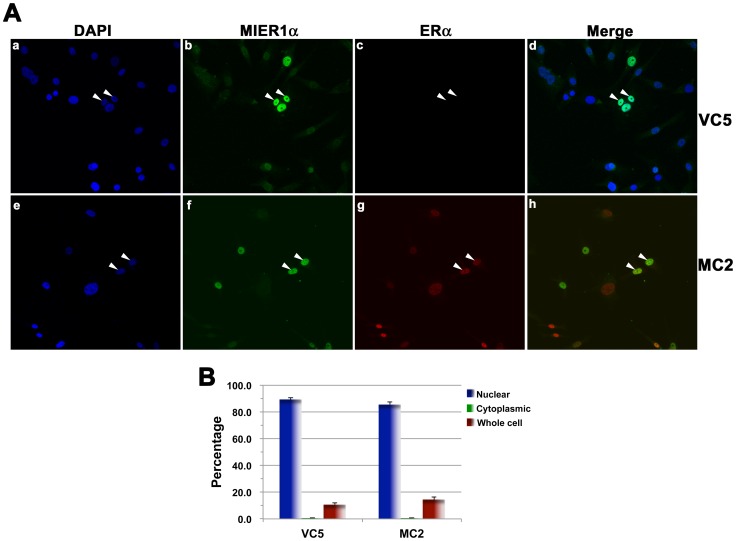
MIER1α is localized in the nucleus in ER- breast carcinoma cells. MDA231-derived cell lines, VC5 (vector) and MC2 (stably expressing ERα), were transfected with myc-tagged MIER1α and analyzed by confocal microscopy using DAPI (a, e), 9E10 anti-myc tag (b,f), anti-ERα (c,g) and the secondary antibodies described in the legend to Fig. 1. Panel d shows merged MIER1α and DAPI staining while panel h shows merged MIER1α and ERα staining. Note that MIER1α is localized in the nucleus in VC5 cells, even in the absence of ERα (arrowheads in panels a-d). (B) Histogram showing the results of 3 independent experiments; random fields were selected and the staining pattern of each cell within the field was scored visually. 170-380 cells were scored for each cell line. Plotted is the percentage of cells in each category ± S.D; there is no significant difference between the percent nuclear for the two cell lines (p>0.05).

To confirm that ERα is not required for targeting MIER1α to the nucleus, we examined localization in two clonal lines of MDA-MB-231 (ER-), MC2 and VC5, stably expressing ERα or empty vector, respectively [10], [11]. MC2 and VC5 cells were transfected with myc-tagged MIER1α and localization was determined by confocal microscopy (Fig. 2). Similar localization patterns were seen in the 2 cell lines: most cells exhibited nuclear MIER1α (Fig. 2B; 89% for VC5 and 86% for MC2), regardless of whether ERα was present (Fig. 2A, panels b-c & f-g). Taken together, these data demonstrate that ERα is not involved in transporting MIER1α to the nucleus.

### The ELM2 domain of MIER1α is required and sufficient for targeting to the nucleus

**Figure 3:**
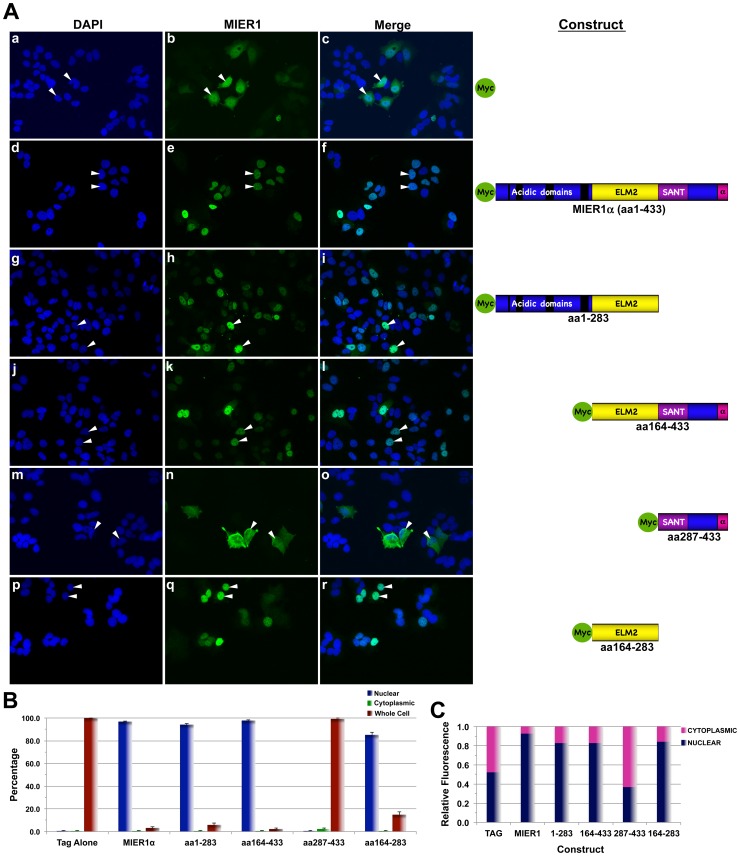
The ELM2 domain is sufficient for nuclear localization of MIER1α. MCF7 cells were transfected with myc-tag empty vector (panels a-c), myc-tagged full-length MIER1α (d-f) or a myc-tagged MIER1α deletion construct containing either the acidic + ELM2 domains (g-i), the ELM2 + SANT + α C-terminus (j-l), the SANT domain + α C-terminus (m-o) or the ELM2 domain alone (p-r). Localization was analyzed by confocal microscopy using DAPI and 9E10 anti-myc tag antibody. (A) Illustrative examples of cells showing stained nuclei and MIER1α localization; arrowheads show examples of nuclei. A schematic, drawn to scale and illustrating the MIER1α domains and constructs used, is shown on the right; the acidic stretches are shown as black bars, the ELM2 domain is in yellow, the SANT domain in purple, the α C-terminus in pink and all remaining sequence in blue. The amino acids (aa) encoded by each construct are indicated. The myc epitope tag is shown in green. (B) Histogram showing the results of 3 independent experiments; random fields were selected and the staining pattern of each cell within the field was scored visually. 220–970 cells were scored for each construct. Plotted is the percentage of cells in each category ± S.D; the percent nuclear for the SANT domain + α C-terminus (aa287-433) construct is significantly less than that for full-length MIER1α (p<0.05). (C) Bar graph showing the intracellular distribution of MIER1α. Pixel values for the nuclear and the cytoplasmic compartments were measured in confocal z-stacks using Image J v1.38; plotted is the proportion of the total signal in each compartment, using measurements from 20–30 cells for each construct. The proportion of the SANT domain + α C-terminus (aa287-433) construct in the nuclear compartment is significantly less than that of full-length MIER1α (p<0.05).

Identifying the region of MIER1α that is required for nuclear targeting might provide insight into the mechanism involved. Therefore, we performed a deletion analysis of myc-tagged MIER1α. MCF7 cells were transfected with plasmids encoding full-length MIER1α (aa1-433) or with a deletion construct containing the following regions: **1**) the N-terminal acidic stretches + the ELM2 domain (aa1-283), **2**) the ELM2 + SANT + α C-terminus (aa164-433), **3**) the SANT + α C-terminus (aa287-433) or **4**) the ELM2 domain alone (aa164-283) (Fig. 3). Localization was determined by confocal microscopy and compared to the myc-tag alone and to full-length MIER1α. The myc-tag alone displays whole cell staining (Fig. 3A, panels a-c; Fig. 3B), as expected of a macromolecule that is sufficiently small (<40 kDa) to undergo passive diffusion through the nuclear pore (reviewed in [15]). Myc-tagged full-length MIER1α, on the other hand, is almost exclusively nuclear (97%; Fig. 3A, panels d-f; Fig. 3B-C). Constructs 1 & 2 localized in the nucleus, similar to full-length MIER1α (94% and 98% nuclear; Fig. 3A, panels g-l; Fig. 3B-C), while construct 3 showed a distribution pattern similar to the myc tag alone, i.e. whole cell (0% nuclear; Fig. 3A, panels m-o; Fig. 3B-C). Thus, only constructs containing the ELM2 domain were targeted to the nucleus and indeed, the ELM2 domain in isolation was localized in the nucleus (85% nuclear; Fig. 3A, panels p-r, & Fig. 3B-C). To obtain a quantitative measure of MIER1α localization within the cell, we performed an analysis of confocal z-stacks for each construct, using the ImageJ software program [12] and determined the fluorescence in the nuclear and cytoplasmic compartments (Fig. 3C). The results of this analysis show that 93% of full-length MIER1α and 83–84% of constructs 1, 2 & 4 are in the nuclear compartment, while only 37% of construct 3 was nuclear. Together these results demonstrate that the ELM2 domain is necessary and sufficient to target MIER1α to the nucleus.

**Figure 4:**
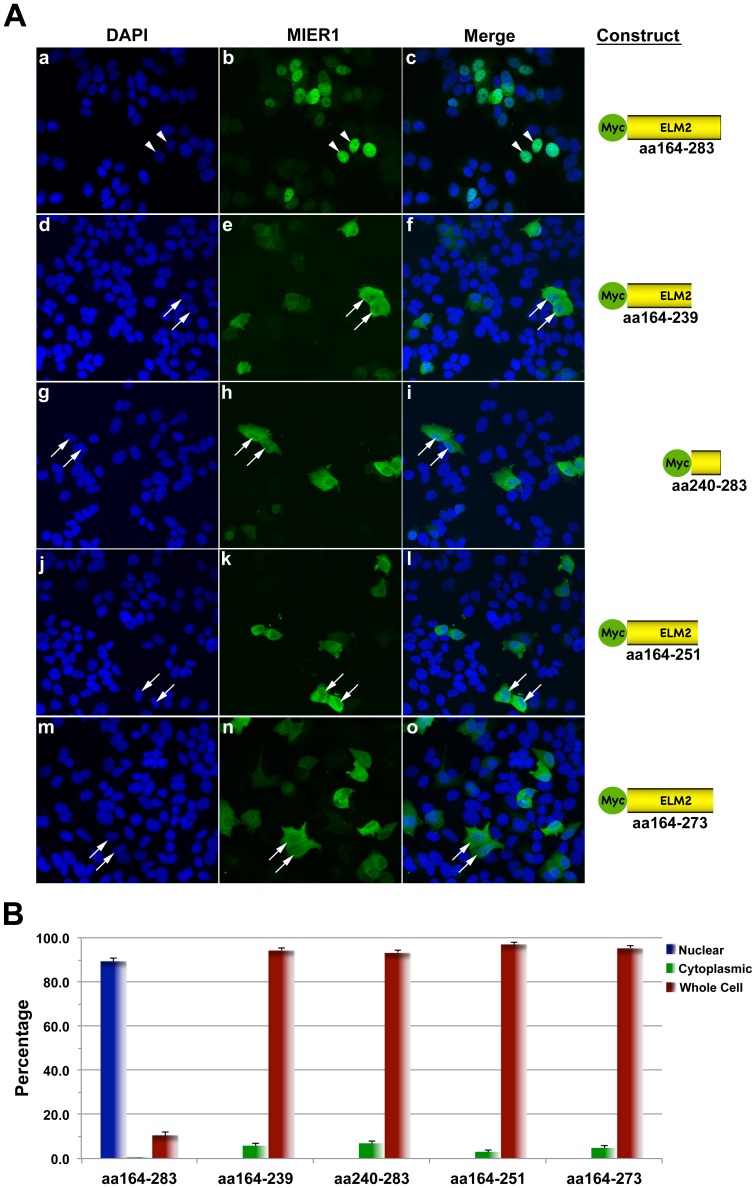
Nuclear localization requires an intact ELM2 domain. MCF7 cells were transfected with a myc-tagged intact ELM2 domain (aa164–283) (panels a-c) or a myc-tagged ELM2 deletion construct containing aa164-239 (panels d-f), aa240-283 (panels g-i), aa164-251 (panels j-l), aa164-273 (panels m-o). Localization was analyzed by confocal microscopy using DAPI and the 9E10 anti-myc tag antibody. (A) Illustrative examples of stained cells showing MIER1α localization. Note that nuclear localization was only detected with an intact ELM2 domain (a-c); arrowheads indicate examples of stained nuclei. The rest of constructs displayed whole cell staining (arrows in d-o). A schematic, drawn to scale and illustrating the constructs used, is shown on the right as are the amino acids (aa) encoded by each construct. The myc epitope tag is shown in green. (B) Histogram showing the results of 3 independent experiments; random fields were selected and the staining pattern of each cell within the field was scored visually. 465-565 cells were scored for each construct. Plotted is the percentage of cells in each category ± S.D; the percent nuclear of all deletion constructs are significantly less than that of the intact ELM2 domain (p<0.05).

To further define the sequence required for nuclear targeting, we produced 4 myc-tagged deletion constructs of the ELM2 domain for analysis. The first two were designed to divide the 120aa ELM2 domain into an N-terminal 76aa and a C-terminal 44aa portion (Fig. 4A, panels d-i). In contrast to the intact ELM2 construct (Fig. 4A, panels a-c, & Fig. 4B), neither portion was targeted to the nucleus (Fig. 4A, panels d-i, & Fig. 4B). To verify that the critical sequence was not bisected in these 2 constructs, we produced 2 additional constructs that maintained the integrity of this region. C-terminal deletions were designed to remove either the last 10aa or the last 32aa. As can be seen in Fig. 4A, panels j-o, & Fig. 4B, neither construct was localized in the nucleus. Thus, removal of as little as 10aa from the C-terminus of the ELM2 domain abolished nuclear targeting. These data lead us to conclude that an intact ELM2 domain is required for nuclear targeting of MIER1α.

### Interaction with HDAC1/2 is required for nuclear localization of MIER1α

**Figure 5:**
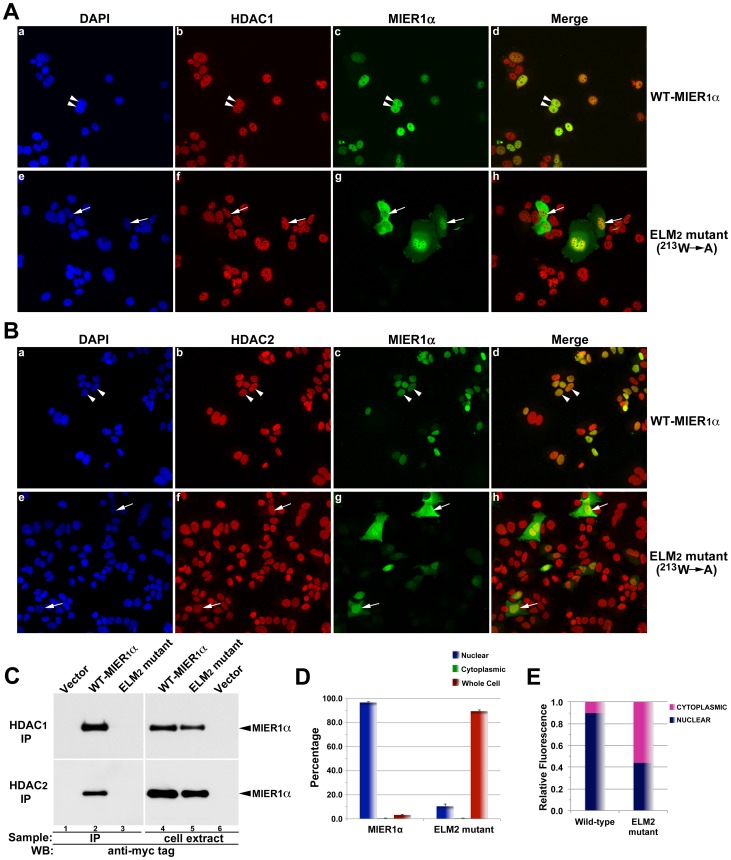
Interaction with HDAC1/2 is required for nuclear localization of MIER1α. MCF7 cells were transfected with myc-tagged, full-length, wild-type MIER1α (a-d) or a full-length, mutant MIER1α (e-h) containing a single point mutation (^213^W→A) in the ELM2 domain (ELM2 mutant) known to abrogate its ability to interact with HDAC1/2. Localization was analyzed by confocal microscopy using DAPI, 9E10 anti-myc tag (AlexaFluor-488) and anti-HDAC1 (panel A) or anti-HDAC2 (panel B) (AlexaFluor-647). (A-B) Illustrative examples of cells showing HDAC and MIER1α localization. Note that the ELM2 mutant loses the exclusively nuclear staining seen with wild-type MIER1α (arrowheads in c) and shows predominantly whole cell staining (arrows in g). (C) Western blot showing that the ELM2 mutant does not interact with HDAC1 or HDAC2. MCF7 cells were transfected myc-tag empty vector (lane 1, 6), myc-tagged full-length wild-type MIER1α (lanes 2, 4) or myc-tagged full-length ELM2 mutant (lanes 3, 5). Cell extracts were either subjected to immunoprecipitation (lanes 1–3) with anti-HDAC1 (upper panel) or anti-HDAC2 (lower panel) or loaded directly on the gel (lanes 4–6). Blots were stained with the 9E10 anti-myc tag antibody. (D) Histogram showing the results of 3 independent experiments; random fields were selected and the staining pattern of each cell within the field was scored visually; >275 cells were scored for each construct. Plotted is the percentage of cells in each category ± S.D; the percent nuclear for the ELM2 mutant is significantly less than that of wild-type MIER1α (p<0.05). (E) Bar graph showing the intracellular distribution of MIER1α. Pixel values for the nuclear and the cytoplasmic compartments were measured in confocal z-stacks using Image J v1.38; plotted is the proportion of the total signal in each compartment, using measurements from 30 cells for each construct. The proportion of the ELM2 mutant in the nucleus is significantly less than that of WT-MIER1α (p<0.05).

The results presented in Figure 4 are reminiscent of a previous study characterizing the interaction of MIER1α with HDAC1 [2]. Utilizing a similar deletion analysis, this interaction was shown to require an intact ELM2 domain. In fact a single point mutation of a highly conserved tryptophan (W) at position 213 in the ELM2 domain abolished interaction between MIER1 and HDAC1. MIER1 also interacts with the highly related HDAC2, but not with any of the other class I, IIa, IIb or IV HDACs [16], [17] and HDAC1/2 are the only proteins known to interact with the ELM2 domain of MIER1α. Therefore, we investigated whether interaction with HDAC1/2 plays a role in nuclear localization of MIER1α. MCF7 cells were transfected with either a myc-tagged, full-length wild-type MIER1α (WT-MIER1α) or a myc-tagged full-length mutant containing the point mutation ^213^W→A (ELM2 mutant) and analyzed by co-IP for interaction with endogenous HDAC1 or HDAC2. Subcellular localization was determined by confocal microscopy in parallel samples. Our co-IP results confirm WT-MIER1α interaction with both HDAC1 and HDAC2 (Fig. 5C, lane 2, upper and lower panels) and demonstrate that the ELM2 mutant does not interact with either HDAC1 or HDAC2 (Fig. 5C, lane 3, upper and lower panels). Confocal analysis revealed that HDAC1 and 2 expression levels were not affected by expression of the ELM2 mutant (Fig. 5A&B, compare panels b and f); however nuclear targeting is lost with this ELM2 point mutation (Fig. 5A&B, compare panels c and g; Fig. 5D), with only 10% of cells now showing nuclear staining. Quantitative analysis of the fluorescence in the nuclear and cytoplasmic compartments using ImageJ shows that 90% of wild-type MIER1α is in the nucleus but that there was significantly less (44%) of the ELM2 mutant located in the nuclear compartment (Fig. 5E; p<0.05). These data suggest that interaction with HDAC1/2 is required to target MIER1α to the nucleus.

**Figure 6:**
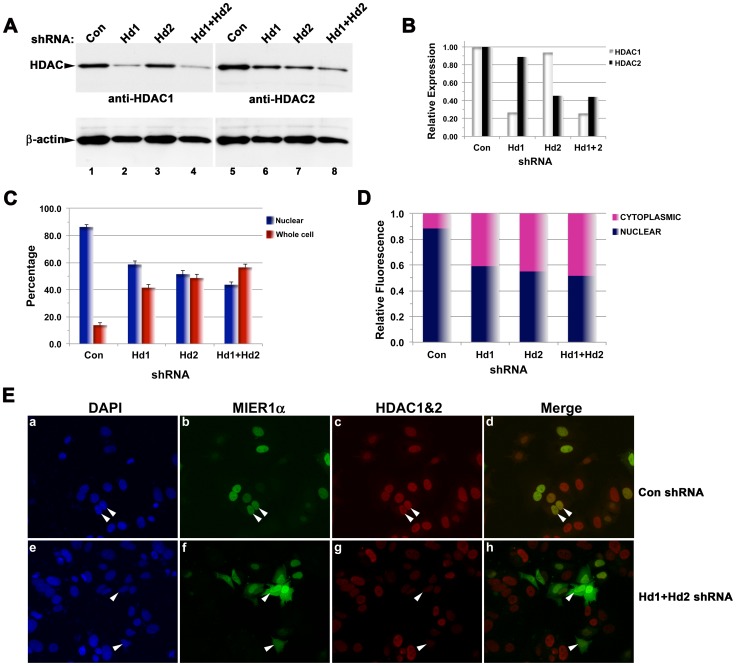
HDAC1 and 2 knockdown reduces nuclear localization of MIER1α. MCF7 cells were transfected with myc-tagged MIER1α and either control shRNA or HDAC shRNA(s), then analyzed by immunoblotting (A–B) or confocal microscopy (C–E). (A–B) Western blot analysis to confirm HDAC knockdown and specificity of each shRNA. Extracts from MCF7 cells were transfected with myc-tagged MIER1α and either control scrambled shRNA (Con; lane 1, 5), HDAC1 shRNA (Hd1; lane 2, 6), HDAC2 shRNA (Hd2; lane 3, 7) or both HDAC1 and 2 shRNAs (Hd1+Hd2; lanes 4, 8). Duplicate samples were stained with anti-HDAC1 (lanes 1–4) or anti-HDAC2 (lanes 5–8). The blots were restained with anti-β-actin (lower panels) to verify equal loading. (B) The HDAC protein bands shown in (A) were quantified using ImageJ, normalized to β-actin and plotted as a proportion of the HDAC level in control cells. Note that each shRNA is specific for its target. (C) Localization was analyzed in parallel samples by confocal microscopy. Histogram showing the results of 2 independent experiments; random fields were selected and the staining pattern of each cell within the field was scored visually; 400–600 cells were scored for each shRNA. Plotted are the percentage of cells in each category ± S.D; the percent nuclear of HDAC1, HDAC2 or HDAC1&2 depleted cells were significantly less than that of controls (p<0.05). (D) Bar graph showing the intracellular distribution of MIER1α. Pixel values for the nuclear and the cytoplasmic compartments were measured in confocal z-stacks using Image J v1.38; plotted is the proportion of the total signal in each compartment, using measurements from 20–25 cells for each shRNA. The proportion of MIER1α in the nucleus is significantly less in depleted cells than in controls (p<0.05 for each). (E) Illustrative examples of cells depleted for both HDAC1 and 2, stained as described in the legend to Fig. 5 for MIER1α (panels b, f) and with combined anti-HDAC1 and 2 antibodies (panels c, g), using MCF7 cells co-transfected with myc-tagged MIER1α and either control shRNA (panels a-d) or HDAC1 + HDAC2 shRNAs (panels e-h). Note that MIER1α staining is nuclear in control cells (arrowheads in a–d) but predominantly ‘whole cell’ in cells with reduced HDAC1&2 staining (arrowheads in e–h).

To confirm the role of HDAC1/2 in nuclear localization of MIER1α, we investigated the effect of depleting HDAC1 and 2 using shRNA. MCF7 cells were co-transfected with a plasmid encoding a myc-tagged MIER1α along with either a control shRNA, an HDAC1 shRNA, an HDAC2 shRNA or both HDAC1&2 shRNAs. Localization was determined by confocal microscopy and quantified by ImageJ analysis of confocal z-stacks (Fig. 6C–E); HDAC1 and 2 knockdown was verified in parallel samples by Western Blot (Fig. 6A–B). Individual knockdowns of HDAC1 and HDAC2 help confirm that each shRNAs used in this analysis is specific for its target and allow us to determine the requirement of each for nuclear localization of MIER1α. HDAC1 shRNA was effective in knocking down endogenous HDAC1 to 27% of control while having little effect on HDAC2 expression (Fig. 6A, lanes 2 & 6; Fig. 6B). Likewise, HDAC2 shRNA reduced endogenous HDAC2 levels to 45% of control without affecting HDAC1 (Fig. 6A, lanes 7 & 3; Fig. 6B). In cells transfected with both shRNAs, HDAC1 and 2 were reduced to 26% and 44%, respectively (Fig. 6A, lanes 4 & 8; Fig. 6B). These data confirm the specificity and effectiveness of the shRNAs used in this set of experiments.

Confocal analysis of cells depleted for HDAC1, HDAC2 or for both revealed a significant reduction in the percentage of cells with nuclear MIER1α when compared to controls (p<0.05; Fig. 6C and 6E, compare panels b&f). Exclusively nuclear MIER1α was detected in 86% of control cells, but reduced to 58% of those depleted for HDAC1, 51% of those depleted for HDAC2 and 44% of those depleted for both (Fig. 6C). Quantitative analysis of confocal z-stacks revealed a similar pattern: in the control, 88% of MIER1α was in the nuclear compartment and this was reduced to 59%, 55% and 52% in HDAC1, HDAC2 and HDAC1&2 depleted cells, respectively (Fig. 6D). These data confirm that depletion of HDAC1 or HDAC2 or both results in a significant reduction of MIER1α in the nucleus (p<0.05). Together, these results demonstrate that both HDACs are involved in targeting MIER1α to the nucleus.

## Discussion

Although small proteins can passively diffuse through the nuclear pore, the majority of proteins with nuclear functions undergo active transport into the nucleus (reviewed in [18]). The most common transport mechanism involves recognition of a classic NLS within the cargo protein by the importins, which then mediate interaction with the nuclear pore complex (NPC) and translocation into the nucleus. However, other mechanisms have been described, including direct binding to nucleoporins in the NPC, e.g. β-catenin [19], piggyback through interaction with another nuclear protein, e.g. LEF-1 [20], BRCA1 (reviewed in [21]) or calmodulin-mediated nuclear import, e.g. Sox-9 [22]. Therefore, it is not surprising that MIER1α can be localized in the nucleus even though it does not contain a recognizable, functional NLS. However, it was unexpected to discover that even though MIER1α binds ERα and inhibits its growth stimulating activity, this interaction is not involved in transporting MIER1α to the nucleus. This leads us to conclude that MIER1α only interacts with ERα once it is in the nucleus.

HDAC1 and 2 are widely expressed [23], [24] and frequently located together in three major multiprotein corepressor complexes: Sin3, NuRD, and CoREST [17], [25], [26]. Interestingly, MIER1 is not contained in any of these complexes, but rather forms part of a unique corepressor complex with HDAC1&2, CDYL and G9a [16], [17]. HDAC1 and HDAC2 are nearly identical [26], [27], with an overall sequence identity of 82% and both belong to the class I HDACs along with HDAC3 and 8 (reviewed in [24]). They contain a C-terminal NLS and, unlike other classes, members of this class are found almost exclusively in the nucleus. HDAC1 can associate with itself as well as heterodimerize with HDAC2 and this interaction is mediated through an N-terminal region that includes part of the conserved HDAC domains [24]. While HDAC's primary role is in chromatin remodeling, HDAC2 has been shown to interact with the endosomal protein APPL1 and contribute to its nuclear localization [28]. Our results provide additional evidence that HDACs can play a role in nuclear localization.

Our data show that depletion of either HDAC1 or HDAC2 reduces nuclear localization of MIER1α, demonstrating that both are involved in this process. It was interesting to note that the reduction in nuclear localization was similar whether HDAC1 or 2 or both were knocked down. This combined with the fact that 80–90% of HDAC1 and 2 exist as heterodimers in MCF7 cells [29], suggests that it is the heterodimer that is required for targeting MIER1α to the nucleus.

In a recent report, we showed that alternative splicing of MIER1α to include an additional exon encoding a functional NES resulted in shuttling of this α isoform to the cytoplasm [7]. Thus RNA splicing may represent a primary mechanism for regulating the nucleo-cytoplasmic distribution of the α isoform. However, we cannot rule out the possibility that the MIER1α isoform is also shuttled out of the nucleus through interaction with a NES-containing protein. MIER1α has been shown to interact several molecules in addition to ERα [8] and HDAC1/2 [2]; these include the histone methyltransferase G9a [30], the chromodomain-containing protein CDYL [31] and the histone acetyltransferase CBP [3]. However, none of these has been reported to contain a NES.

Current evidence suggests that MIER1α functions as a tumour suppressor [8], possibly through its interaction with ERα. Our previous analysis of normal breast tissue and breast cancer tumours using an antibody that specifically recognizes the α C-terminus, showed that the α isoform(s) is localized in the nucleus in normal tissue and in hyperplasia, however the percentage of cells with nuclear staining decreased to 50% in DCIS and to 4% in IDC [8]. This suggests that loss of nuclear MIER1α might represent a critical event in breast cancer progression since shuttling to the cytoplasm would interfere with its nuclear function as a transcriptional repressor. It is also possible that MIER1α has additional, as yet undescribed, activity in the cytoplasm. Several instances of dual roles have been reported for other transcriptional regulators (reviewed in [32], [33]). For example, ERα functions in the nucleus to regulate transcription of target genes but also has non-genomic functions (reviewed in [34]). Most of these involve activation of various signaling cascades in a tissue-specific manner, including activation of ERK, PI3’K and Akt pathways as well as signaling through GPCR and growth factor receptors. Whether or not MIER1α also has non-genomic functions awaits further investigation.
